# How will the COVID-19 pandemic shape the future of meat consumption?

**DOI:** 10.1017/S136898002000316X

**Published:** 2020-12

**Authors:** Sophie Attwood, Cother Hajat

**Affiliations:** 1 Independent Scholar; 2 Public Health Institute, UAEU, Al Ain, United Arab Emirates

**Keywords:** Nutrition, Sustainability, Diet, COVID-19

## Abstract

Since its recent onset, the COVID-19 pandemic has altered the daily lives of millions around the world. One area particularly affected is our diets, with food supply chain disruptions, media coverage of food safety issues and restaurant closures all influencing consumer dietary behaviour. Given this situation, we pose a timely question – what is the impact of the current pandemic on longer-term meat consumption patterns? This issue is pertinent given accumulating evidence that overconsumption of meat, particularly red meat, is associated with negative environmental and health outcomes. Here, we discuss how the current pandemic has already begun to shift public awareness of illnesses linked to animals and has resulted in short-term changes in patterns of meat consumption. Past zoonotic outbreaks, such as SARS and swine flu, are also referred to, and we find that these led to similar short-term reductions in meat intake, a shift in the type of meat chosen and longer-lasting impacts on consumer perceptions of the health risks associated with meat. We conclude that, if immediate changes in eating patterns as a result of COVID-19 are retained in the longer term, one possible opportunity to emerge from the current pandemic may be a shift away from overconsumption of meat, leading to potential health and environmental benefits in the longer term.

## Zoonosis and animal-based foods

The term ‘Zoonosis’ entered our common vocabularies in 2020 with onset of the COVID-19 pandemic. Until this year, the notion that infectious diseases could originate from animals was unknown to many. This is despite recent estimates indicating that about six out of every ten known infectious diseases in humans come from interactions with animals, while three out of every four emerging infections are predicted to be zoonotic in future^(1)^. Such high likelihood of between-species disease transmission poses a considerable threat to human health, wellbeing and economic prosperity, especially given research to suggest that there are about 1·7 million undiscovered viruses currently hosted in wildlife^(2)^. It is for this reason that the WHO is now emphasising the need for countries to adopt OneHealth approaches in their planning and action agendas. This involves experts across a range of sectors working together to promote better public health by focusing on risks that, just like COVID-19, lie at the intersection of human and animal health and the environment^(3)^.

However, it is not just the close link between animals and infectious diseases that people are often oblivious to. For many, the basic notion that many of the foods we commonly eat come from animals is unclear. And who can blame them? Burger patties and battered nuggets are hardly recognisable as parts of cows or chickens, while cuts of meat are given names like ‘steak’, ‘sausage’ or ‘fillet’, dissociating them from their animal source^(4)^. This disconnect is reflected in the findings of a 2017 British Nutrition Foundation survey of 27 000 children in the UK, indicating that misconceptions about food are common. Nearly, a third of those questioned believed cheese came from plants and 14 % were unaware that bacon is part of a pig^(5)^.

## COVID-19 and consumer perceptions of meat

Dissociation between animals and the food they produce reflects modern agricultural and shopping practices. Meat, egg and dairy production is now predominantly managed by large agribusinesses. Consumers tend to only interact with the end product when it is presented, neatly pre-packaged and pre-prepared, on supermarket shelves. The food production process is otherwise entirely opaque, with few shoppers seeking clarity on how and where their food comes from^(6,7)^.

But, this situation might be changing. Given the current pandemic, a pertinent question many are now asking is whether our dietary status quo could be evolving as a result of COVID-19. In particular, might consumer demand for animal source foods decrease, increase or stay the same, and, if such changes are afoot, how long might these last^(8)^?

One reason to believe that demand may be decreasing is to consider the role that the media has played in highlighting the zoonotic root of coronviruses. This has helped to open the eyes of many to just one of the safety issues associated with consuming animals. As a result of extensive media coverage in China, a nationwide ban on using wild animals for food has now been imposed by the Chinese Government^(9)^. Prior to this, markets in China, including the Wuhan Seafood Wholesale Market where the current pandemic is thought to have started, sold an array of wildlife, including porcupines, snakes, rats and the now infamous pangolin^(10)^. While this move was imposed rapidly, commentators have pointed out that similar bans were instituted following previous outbreaks in China (i.e. in response to severe acute respiratory syndrome-related coronavirus (SARS-CoV) in 2003), but, because these were not passed into law, they were eventually relaxed^(11)^.

Could the current outbreak lead to a different regulatory outcome in China this time around? In our increasingly connected world, the difference between 2020 and previous outbreaks is that the impact of the disease has now extended far beyond China’s national borders. As a result, the country has come under substantial international pressure to change how they manage domestic food safety issues. For example, the WHO has recently updated their guidance calling for better regulation of Chinese wet markets, ensuring these conform to food safety and hygiene standards in order to lower future pandemic risks^(12)^. Late in May, the region of Wuhan responded by passing a wet market ban into law in that province, with a proposed duration of 5 years^(13)^.

## Animal agriculture, processing and human health

Beyond China, media reports of the COVID-19 pandemic have stoked interest in meat production practices more generally. Conversations have ignited around the role that factory farming plays in accelerating infectious disease spread^(14)^, with new light shone on one other serious, yet often ignored risk of intensive animal husbandry – antimicrobial resistance^(15)^. Liberal use of antibiotics in animal feed, to manage disease and promote growth, has been recognised as an impending risk to human health for some time^(16)^. Yet, until now, few seemed willing to take decisive action to reduce the potential public health threat that this poses. COVID-19 may have the power to change this, mobilising consumers to demand greater transparency about how their meat is produced, encouraging more individuals (especially in high-income countries) to select organic or grass-fed cuts over cheaper, intensively farmed alternatives, and kick-starting a shift into the rapidly growing ‘meat alternative’ category^(17)^.

Adding to this scrutiny is further media coverage of COVID-19 in meat packaging employees, particularly in the US where an estimated 25 000 have been infected and ninety died as a result of localised factory outbreaks^(18)^. The resultant shutdown of multiple processing sites has not only led to mass culls of animals and a shortage in meat supply in the US (e.g. pork production is estimated to be down 6 % on 2019)^(19)^ but has helped to highlight some of the human aspects associated with systemised slaughter. Workers in meat processing plants are typically low paid, overcrowded and engaged in gruelling and messy work. Adding to this are complaints from employees of insufficient protective clothing and a lack of sanitation options to minimise their infection risk^(20)^. For an industry optimised for efficiency, social distancing measures and potentially costly safety regulations disrupt the smooth running of operations. It remains to be seen whether consumer behaviour will change as a result of these issues coming to light in the media – either in response to potential price increases to compensate for losses in meat sales or from consumers actively boycotting meat to stand in solidarity with workers.

## Disrupted supply and eating habits

A second potential way that COVID-19 may be reducing intake of animal products is through supply chain disruption and panic-buying^(21)^. Revenue from meat sales has reportedly dipped since the onset of the pandemic, attributed in large part to restaurant closures. Meat is often favoured over vegetarian dishes when dining out, an option temporarily paused for many. This has left more expensive cuts of meat unsold and storage facilities at peak capacity^(22)^. So far, lost restaurant sales have yet to be entirely compensated for by increased supermarket spend on meat products^(23)^. Consumers have reportedly been stocking up on longer-lasting shelf staples instead. For example, tinned goods manufacturer Kraft Heinz published a 6 % sales boost during the first quarter of 2020 as consumers purchased cans of soup and baked beans, anticipating 14-day isolation periods and wishing to minimise the frequency of their grocery store visits^(24)^.

In addition to shelf staples, shoppers in high-income countries also appear to be purchasing far more organic products than they were pre-pandemic, with sales in the UK up by more than 25 % in March and April 2020. This is thought to be due, in part, to a desire for assurance that their food is safe and nutritious, as well as a boost in subscriptions to organic box delivery schemes as consumers are keen to avoid escalating supermarket queues due to social distancing regulations^(25)^. Similarly, US sales of plant-based meat alternatives have also reportedly increased, up by almost 200 % in April 2020 compared to the same period in 2018^(26)^. This has been ascribed to a combination of shortages in meat availability, heighted concern about food safety and health^(27)^, plus competitive marketing of meat alternatives as producers seize the current disruption as an ideal opportunity to attract new customers^(28)^.

It is possible that this shift towards consuming more plant-based foods and less dining out could benefit both population health and the environment in high-income countries. The trends that have been observed in response to COVID-19 may lead to lower intakes of salt, energies and saturated fat from food purchased outside the home (i.e. as restaurant meals), while increased purchasing of beans and pulses could lower the environmental footprint of diets if these are consumed instead of meat heavy options rather than in addition to them^(29)^. For such benefits to be fully realised, however, short-term shifts must become ingrained habits that are maintained in the longer term.

## Learning from the past

This leads us to the next question of whether populations around the world will continue to cook at home, eat less meat and more beans when the current pandemic subsides, or whether old patterns will return as life goes back to a ‘new’ normal? To answer this, we can look at the longer-term impact of previous, similar public health crises for answers, specifically those involving diseases originating from animal sources.

Most closely aligned to the current situation is the outbreak of SARS, emerging in Hong Kong in 2003. Research conducted after this outbreak showed increased consumer concern with health, leading to adoption of healthier diets (i.e. avoiding excess sweet and cholesterol-rich food)^(30)^. Equally, following the Avian Influenza outbreak in 2013 in China, decreased sales of poultry were evident up to a year after onset. When questioned as to why their shopping habits had changed, Chinese consumers spoke of their fears about contracting flu from poultry and a lack of trust in institutions to ensure the safety of their diets^(31)^.

More recently, the 2019 African Swine Flu outbreak led to significant disruption in Chinese pork supply, with mass culls reducing availability and pushing up prices as a result. These factors, in combination with safety concerns, led many Chinese consumers to switch to alternative protein sources, reflected in depressed pork sales during 2019, down about 10–15 %^(32)^. African Swine Flu seems to have accelerated a background consumer trend towards more diversified protein intake in China, with consumers trying a wider range of protein sources, like seafood, beef, mutton and poultry^(33)^. Some are now speculating as to whether COVID-19 may have a similar catalytic effect on consumption of plant-based foods in Western nations, with the virus adding momentum to an already rapidly growing trend towards reduced intake of animal-based foods^(34)^.

Previously, the BSE scandal in cattle in the UK during the 1980s and early 1990s also reportedly led to consequences for consumer food choices. Following the discovery of a link between meat from infected cows and Creutzfeldt–Jakob disease, which causes brain damage in humans, beef intake dropped drastically. Research just after this event showed that up to 30 % of consumers reduced beef consumption immediately following the scare^(35)^. Follow-up research in the late 1990s indicated that negative media attention led to sustained losses in beef sales, although BSE seems to have had little impact on intake of other types of meat^(36)^.

Beyond the UK, further research on perceptions of beef also indicates that food scares like BSE can have a longer-term impact on consumer attitudes. One study from Canada shows heightened risk perceptions are evident up to 8 years after the first disease outbreak^(37)^. While risk perceptions are by no means the sole determinant of meat consumption, with consumers valuing taste, price and convenience above ethical or health concerns^(38)^, this research does highlight the far-reaching impact that media coverage of food-related risks can have on consumer behaviour.

## Conclusion

The COVID-19 pandemic has already increased public awareness of zoonoses and led to short-term modifications in meat consumption. Past zoonotic outbreaks such as SARS and swine flu, albeit on a much smaller scale and far less global, led to short-term reductions in meat consumption, a shift towards certain types of meat and a more pervasive change in perceptions of the health risks from meat consumption. If previous behavioural patterns reoccur, a possible consequence of the COVID-19 pandemic may be to catalyse the shift towards lower meat diets that we are beginning to see in some high-income countries. As with many aspects of this pandemic, we must watch to wait and see.
